# Adult co-creators’ emotional and psychological experiences of the co-creation process: a Health CASCADE scoping review protocol

**DOI:** 10.1186/s13643-024-02643-9

**Published:** 2024-09-11

**Authors:** Lauren McCaffrey, Bryan McCann, Maria Giné-Garriga, Qingfan An, Greet Cardon, Sebastien François Martin Chastin, Rabab Chrifou, Sonia Lippke, Quentin Loisel, Giuliana Raffaella Longworth, Katrina Messiha, Mira Vogelsang, Emily Whyte, Philippa Margaret Dall

**Affiliations:** 1 School of Health and Life Sciences, Glasgow Caledonian University, Glasgow, UK; 2 Faculty of Psychology, Education and Sport Sciences Blanquerna, Universitat Ramon Llull, Barcelona, Spain; 3 Department of Community Medicine and Rehabilitation, Umeå University, Umeå, Sweden; 4 Department of Movement and Sports Sciences, Ghent University, Ghent, Belgium; 5 Department of Psychology and Methods, Jacobs University Bremen, Bremen, Germany; 6 Department of Public and Occupational Health, Amsterdam UMC, Amsterdam Public Health Research Institute, Vrije Universiteit Amsterdam, Amsterdam, The Netherlands

**Keywords:** Co-creation experience, Emotion, Psychological response, Adults, Scoping review

## Abstract

**Background:**

There is a growing investment in the use of co-creation, reflected by an increase in co-created products, services, and interventions. At the same time, a growing recognition of the significance of co-creators’ experience can be detected but there is a gap in the aggregation of the literature with regard to experience. Therefore, the purpose of this scoping review is to uncover the breadth of existing empirical research on co-creation experience, how it has been defined and assessed, and its key emotional and psychological characteristics in the context of co-created products, services, or interventions among adults.

**Methods:**

The development of the search strategy was guided by the research question, Arksey, and O’Malley’s scoping review methodology guidelines, and through collaboration with members of the Health CASCADE consortium. The results of the search and the study inclusion process will be reported in full and presented both narratively and by use of the Preferred Reporting Items for Systematic Reviews and Meta-analyses extension for scoping review (PRISMA-ScR) flow diagram. Comprehensive searches of relevant electronic databases (e.g. Scopus) will be conducted to identify relevant papers. Snowball searches to identify additional papers through included full-text papers will be done using the artificial intelligence tool, namely, Connected Papers. All review steps will involve at least two reviewers. Studies in English, Dutch, Chinese, Spanish, and French, published from the year 1970 onwards, will be considered. Microsoft Excel software will be used to record and chart extracted data.

**Discussion:**

The resulting scoping review could provide useful insights into adult co-creators’ experience of participating in the co-creation process. An increased understanding of the role of emotional and psychological experiences of participating in co-creation processes may help to inform the co-creation process and lead to potential benefits for the co-creators and co-created outcome.

**Systematic review registration:**

10.5281/zenodo.7665851.

**Supplementary Information:**

The online version contains supplementary material available at 10.1186/s13643-024-02643-9.

## Background

Co-creation can be defined as “any act of collective creativity that involves a broad range of relevant and affected actors in creative problem-solving that aims to produce a desired outcome” [1]. Co-creation is increasingly acknowledged as a promising approach to address complex ‘wicked’ societal problems and develop more contextually relevant interventions to improve outcomes in a variety of settings [2]. By facilitating communication across sectors, integrating diverse forms of knowledge and expertise, and enabling local ownership, co-creation can be useful in a broad range of fields including, healthcare, community, and education [3].

The co-creation process is guided by participatory methodologies [4]. The goal of participatory research is to engage all those who are the subject of the research in all stages of the research [5]. Participatory research acknowledges the value of their contribution in a practical and collaborative way [5]. Co-creation builds on these participatory methodologies, to address the power imbalances stemming from social inequities and uses empowerment approaches to address and meet the needs of citizens [3]. Co-creation is more specific than the broad concept of participation, which also refers to passive involvement [6]. The ultimate goal of co-creation is to actively involve all relevant and affected stakeholders in all aspects of the co-creation process, such as planning or conducting [7].

Whilst the co-creation behaviour of participants in a co-creation process is mostly documented in the co-creation literature, the emotional and psychological experience of participating in the co-creation process has been given less attention [8, 9]. Co-creation behaviour is argued to comprise multiple behavioural dimensions that fall under two higher-order factors, namely, participation behaviour and citizenship behaviour [10]. The behavioural dimensions of participation behaviour include information seeking and sharing, responsible behaviour, and personal interaction. The dimensions of citizenship behaviour include feedback, advocacy, helping, and tolerance [10]. On the other hand, the co-creators’ experiences of participating in the co-creation process, hereby shortened to co-creation experience, capture co-creators’ emotional and psychological states; highlight the interactive component; and involve a continuous process as opposed to a single fixed-time event [9]. In brief, the co-creation experience, as defined for the purposes of this review, is the co-creators’ emotional and psychological states during active participation and interaction when engaging in the co-creation process [9]. Co-creation experience differs from co-creation behaviour due to its focus on the feelings and cognitions derived from the act of undertaking the co-creation behaviour [9].

Research indicates that active involvement in the co-creation process can have profound positive effects on increased health and performance outcomes, satisfaction, and well-being [11, 12]. For example, Leask et al. [13] reported older adults having positive experiences engaging with the co-creation of a health intervention, describing that participants’ role as co-researchers made it enjoyable, interesting, and rewarding. Similar findings from Rooijen et al. [14] indicated that participants felt empowered, liked the interactive characteristic of meetings, and felt they were valued contributors with a shared responsibility for the project. Positive emotional states like happiness or gratitude can foster trust, which is important for building relationships, whereas negative emotional states, like anger, uncertainty, and frustration, can decrease trust [15]. Building relationships is an important aspect of the co-creation process, in which experiencing positive emotions helps to create new relationships [16]. Therefore, positive emotions could also contribute to the functioning of the co-creation group(s) and the successful development of products like intervention components, tools, and further actions.

**Fig. 1:**
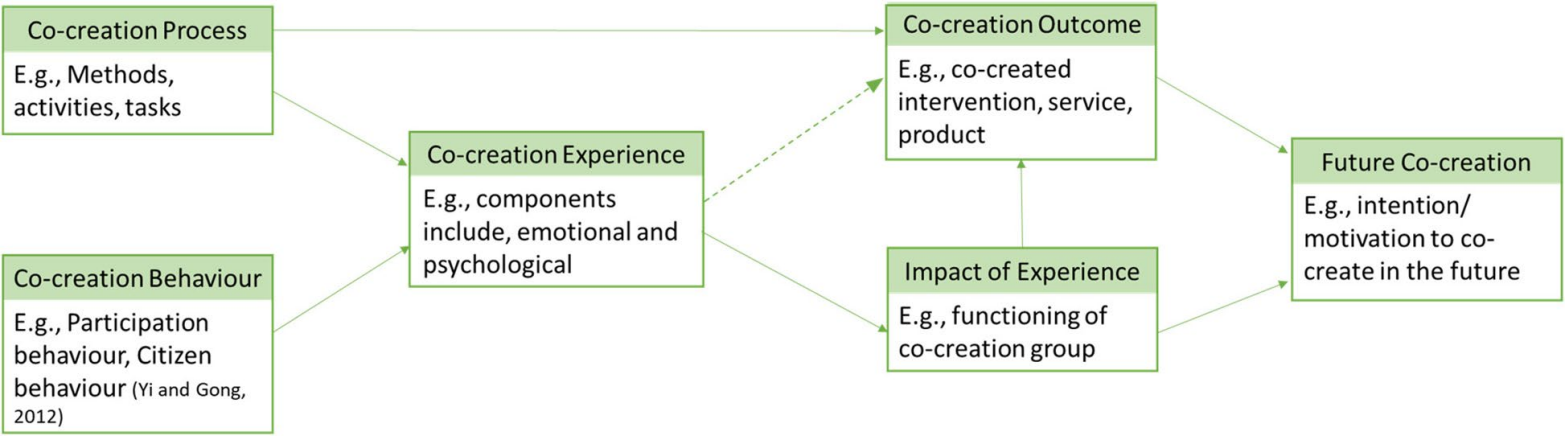
Suggested model of the relationship between co-creation experience, processes, behaviour, outcomes, impact, and future co-creation

There are instances when co-creators can experience the co-creation process negatively. There exists some research to indicate how failed co-created services recovered can impact co-creators in terms of future intention to co-create, role clarity, and motivation [17]. However, there might be a lack of, or a lack of visibility of, literature documenting the negative emotional and psychological experiences associated with the co-creation process because of publication bias. Individual and interpersonal experience including group dynamics are central to the creation of value and innovation and this justifies the need to study the role of human experience in the context of co-creation [18, 19]. Figure 1 provides a visual depiction of the proposed connection between co-creation experience and the other elements of co-creation.

However, so far, there is a gap regarding the aggregation of the literature pertaining to co-creation experience. Therefore, the purpose of this scoping review is to uncover the breadth of existing empirical research on co-creation experience, how it has been defined, and assessed and its key characteristics in the context of co-created products, services, or interventions among adults. As the focus is on the participant’s experience of the process and not the outcome, no limits have been applied to the co-creation context. Scoping reviews are exploratory in nature and systematically map available literature on a broad topic to identify key concepts, theories, sources of evidence, and research gaps [20]. A scoping review has been identified as an appropriate means to address this broad research question given that, to the authors’ knowledge, there has been no systematic review of co-creation experience literature, the phenomenon is not well understood or utilised, and studies span a wide variety of fields. The aim of the current scoping review is to deliver an evidence-based review of co-creators’ experiences of co-creating. This review will guide future research to advance evidence-based co-creation methods and inform guidance aimed at enhancing positive experiences for those participating in co-creation.

### Research question

What is the current state of the science regarding adult co-creators’ emotional and psychological experiences of participating in co-creation?

### Objectives

The objectives of this review are to:

1. Determine the extent of research on co-creation experience.
2. Uncover the range of and key characteristics of emotional and psychological experiences documented in the literature to date.
3. Identify any explicit or implicit underlying psychological theories drawn upon to explain the potential mechanism of the experience of co-creation.
4. Document any tools or technology used during the co-creation process that impacted the experience during co-creation or to make co-creation more successful*.*

## Methodology

This scoping review protocol is reported in accordance with the Preferred Reporting Items for Systematic Reviews and Meta-Analyses Protocols (PRISMA-P) checklist (see Additional file 1).

### Search strategy

**Fig. 2:**
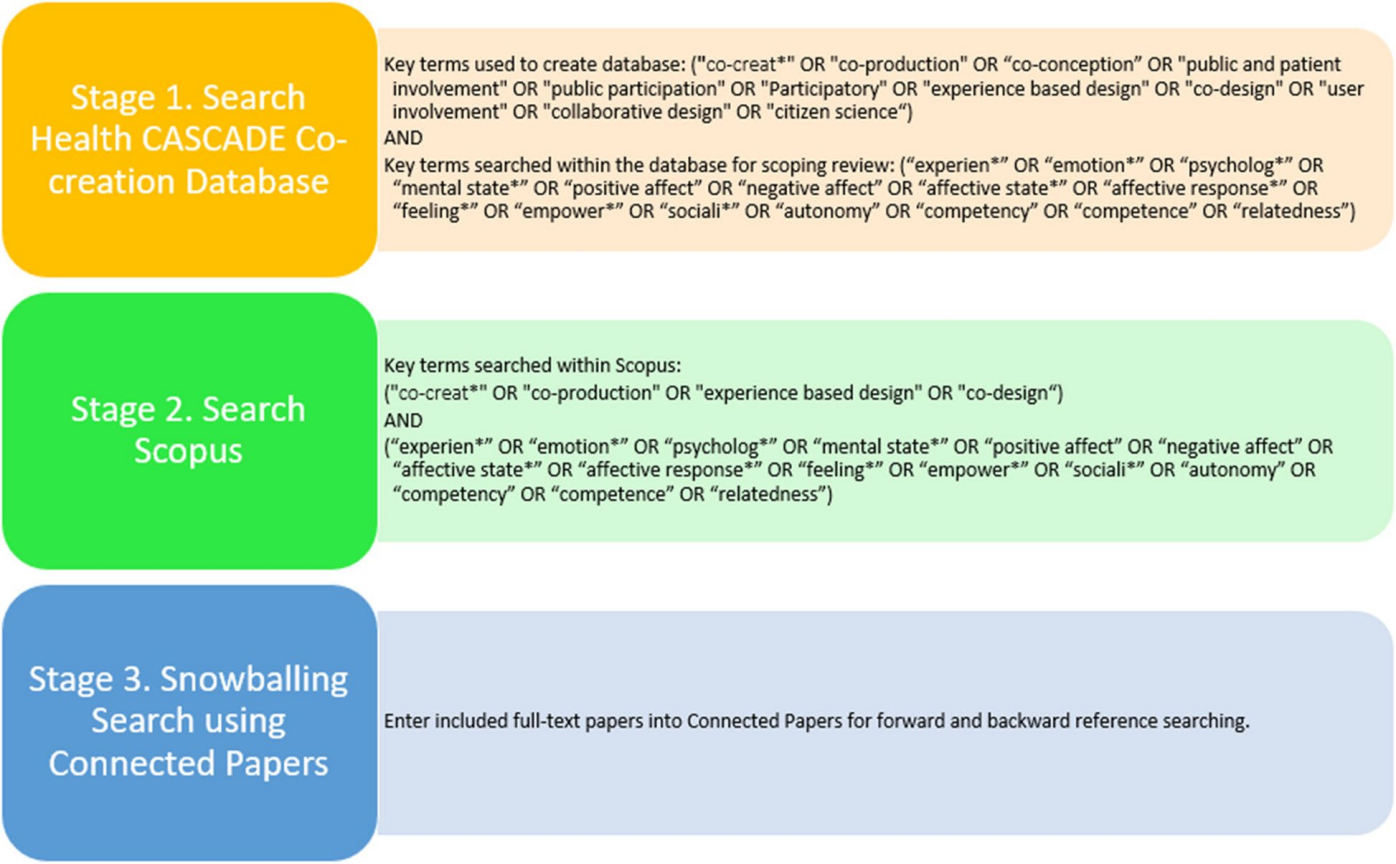
Stages of search strategy

The search strategy comprises three main stages (see Fig. 2). The first stage involved searching the newly created Health CASCADE Co-creation Database. This database was created by members of the Health CASCADE network and was aimed at collecting in one place the entire corpus of literature pertaining to participatory research and co-creation (1). This database was created using CINAHL, PubMed and all databases accessible via ProQuest through Glasgow Caledonian University (GCU) institutional licence (17 databases in total, APA PsycArticles®, APA PsycInfo®, Art, Design & Architecture Collection, British Periodicals, Coronavirus Research Database, Early Modern Books, Ebook Central, Entertainment Industry Magazine Archive, Humanities Index, Periodicals Archive Online, ProQuest One AcademicTrial-Limited time only, PTSDpubs, SciTech Premium Collection, Social Science Premium Collection, Sports Medicine & Education Index, The Vogue Archive, and The Women's Wear Daily Archive). The key search terms used in this search strategy are found in Table 1. ASReview, an artificial intelligence (AI) aided platform that helps find relevant records was used for screening the records to be included in this database. The AI performs a textual analysis of the provided records, based on active learning and prioritization. Given the large volume of records retrieved from PubMed, CINAHL, and all databases available through ProQuest with GCU access, AI was necessary to speed up the screening process. There are over 13,000 records contained in this database, with all titles and abstracts containing at least one of the search terms.

**Table 1:** Key search terms used to capture co-creation

Co-creation	
"co-creat*" OR "co-production" OR “co-conception” OR "public and patient involvement" OR "public participation" OR "Participatory" OR **"experience based design" OR "co-design"** OR "user involvement" OR "collaborative design" OR "citizen science"	

Search terms in bold are the terms selected for use in Scopus

The Health CASCADE Co-creation Database was searched using free-text terms relating to co-creation experience (see Table 2). Search terms have been developed in reference to the research question and through consultation with members of the Health CASCADE consortium. The search will be piloted to check the appropriateness of keywords and to ensure known studies are identified.

**Table 2:** Search terms used to capture the co-creation experience

Co-creation experience	
“experien*” OR “emotion*” OR “psycholog*” OR “mental state*” OR “positive affect” OR “negative affect” OR “affective state*” OR “affective response*” OR “feeling*” OR “empower*” OR “sociali*” OR “autonomy” OR “competency” OR “competence” OR “relatedness”	

The second stage of the search strategy is to use both sets of search terms (see Tables 1 and 2) in Scopus using the Boolean operator AND to combine the two sets. This is to provide additional robustness to the search. Due to the large volume of records retrieved (> 35,000) when combining the two sets of search terms, it is necessary to omit some search terms used to create the Health CASCADE Co-creation Database. Four search terms will be retained “co-creat*”, “co-production”, “co-design” and “experience-based design”. These search terms are specifically chosen because co-production and co-design are commonly used interchangeably with the term co-creation [21]. In addition, “experience-based design” is retained due to the obvious focus on the experience. We will include articles that meet our inclusion criteria for co-creation, regardless of the terminology used to describe the methodology. For pragmatic reasons, sources of unpublished empirical studies (including grey literature, theses, and dissertations) will not be searched for. The draft search strategy for Scopus is available in Additional file 2.

The final stage of the search is to employ snowballing to capture any additional articles that may be potentially missed. An artificial intelligence tool called Connected Papers [22] will be used to identify papers that (1) the included paper has cited (backward reference searching), and (2) papers that have since cited the included paper (forward reference searching).

The article selection process is considered an iterative process, whereby the search strategy will be initially broad and then refined based on abstracts retrieved and as reviewer familiarity with the literature increases. The concept of co-creation is defined differently depending on the setting and context and is often used interchangeably with similar, yet distinct concepts, but equally lacking a clear universal understanding [21]. Therefore, to account for the overlaps in terminology a broad scope will be initially implemented.

As recommended by Arksey and O’Malley [23], decisions on how to set search parameters will be made after a general scope of the field has been gained. Hence, this stage will require the reviewer(s) to engage in a reflexive way and repeat steps to ensure a comprehensive literature search with more sensitive searches [23, 24].

### Inclusion/exclusion criteria

- All study participants in the included papers must be adults, described as people aged 18 years and over with no upper limit. Children/adolescents are not included in this study as research indicates that there are differences between their emotional experiences in terms of emotional intensity and stability [25].
- Empirical articles (i.e. primary research studies) include any qualitative, quantitative, and mixed-method research designs that include a description of the co-created product, service, or intervention and an evaluation of the co-creators’ co-creation experience. Although scoping reviews can draw on evidence from non-empirical sources, this review imposes limits to include empirical sources only as empirical sources would be most useful and appropriate for contributing to an evidence-based understanding of co-creation methods.
- Any context that involves the co-creation of a product, service, or intervention will be considered.
- The Health CASCADE Co-creation Database is limited to searching records between 1st January 1970 and 1st December 2021. The search in Scopus will include records from 1st January 1970 until the date of the search.
- The Health CASCADE Co-creation Database is limited to only include materials that are written in English. However, for the search conducted in Scopus, publications in English, Spanish, Dutch, French, and Chinese languages will also be considered, as the research team has proficient fluency in these languages.

### Data extraction

Following the database search, articles will be exported as a CSV file for removal of duplicates in Excel. The articles will be imported and screened in Rayyan. The title and abstract of all studies will be screened independently by several reviewers (LMcC, QA, QL, EW, GRL, RC, and MV) and irrelevant studies will be removed. All titles and abstracts will be double-screened. Full-text articles of studies identified as potentially relevant for inclusion will subsequently be sought and screened by several reviewers (LMcC, QA, QL, EW, GRL, RC, MV, and KM) against the agreed set of criteria. Differences of opinion regarding inclusion or exclusion will be resolved by discussion and reaching a consensus or by a third reviewer. The results of the search and the study inclusion process will be reported in full in the final scoping review and presented both narratively and by use of the Preferred Reporting Items for Systematic Reviews and Meta-analyses extension for scoping review (PRISMA-ScR) flow diagram.

To determine the extent of research on co-creation experience (objective 1), details about co-creation more generally will first be extracted. This includes:

- Study’s definition of co-creation and co-creation experience (if available).
- The context or setting.
- Data about the participants (number, type, and characteristics of co-creators’ involved).
- Description of the co-creation process undertaken (including number of sessions, level of participation).
- Purpose of co-creation.
- Outcome of the co-created intervention, service, or product.

The key characteristics of psychological and emotional experience including positive and negative components (objective 2) will be extracted.

The psychological theory underpinning the co-creation experience identified by the authors of the studies (objective 3) will be recorded.

Information about the technology or tools that had an impact on the co-creation experience (objective 4) will be extracted.

Additional descriptive information such as discipline and date of publication will also be extracted.

The above-extracted information will be entered into an Excel spreadsheet developed by the authors. This data extraction Excel spreadsheet may be modified and revised as necessary during the process of extracting data from the included evidence sources to ensure that key findings relevant to the review question are addressed.

### Quality assessment

There exists debate as to whether a scoping review should contain an assessment of study quality [26]. A quality assessment component will be included in this review in relation to the sufficiency of reporting the process of co-creating an intervention, service, or product. This tool (see Table 3) has been adapted from Leask et al.’s [4] ‘checklist for reporting intervention co-creation’ and Eyles et al.’s [27] amended version of a checklist for reporting non-pharmacological interventions. The reason for including this checklist is two-fold. Firstly, the scoping review may contain a variety of study designs and the focus is not solely on the outcomes, but rather on the process [27]. Secondly, as explained above, the concept of co-creation is used interchangeably with other similar overlapping concepts, such that some processes may be described as co-creation when they are in fact not (according to the definition used in this review) or vice versa. Therefore, by incorporating this checklist, it will become clearer as to the type or extent of co-creation processes that were implemented and whether they were clearly reported within each individual source of empirical evidence. However, given that a scoping review aims to present an overview of the extant literature on a particular topic without synthesis from individual studies, no study will be excluded on the basis of the quality of reporting co-created interventions.

**Table 3:** Checklist for sufficiently reporting co-created intervention, service, or product

Section	Checklist item	Response (yes; no; partly)
Planning		
	Was the sampling procedure described? (criteria, setting of recruitment)	
	Was it clear where the co-creation of the intervention, service, or product took place? (online, onsite)	
	Was a clear description of the co-creators provided? (demographic information, number, characteristics of interest)	
	Was it clear who facilitated the co-creation process?	
Conducting		
Procedure components	Was there evidence of an attempt to manifest ownership? (branding of the group, identifying rights and responsibilities)	
	Was the level of participation from the co-creators described? (equal, decision power, all stages)	
	Was the overall aim of meetings and the purpose of each meeting presented to the group?	
Procedure methods	Was the frequency of meetings described? Was the duration of the meetings described?	
	Were any interactive techniques and materials used in the co-creation process adequately described?	
	Was the description of the overall co-creation process complete?	
Evaluation		
Process	Was co-creator satisfaction, experience or contribution evaluated? (retention rates)	
	Were the results reported back to the co-creators and public?	
Outcome	Was the outcome of the intervention, service or product described?	
	Were plans for formal testing of the effectiveness or scalability of the co-created intervention, service or product discussed?	
	Was there an explanation of how the validity of the process and outcome were evaluated? (face validation)	

Adapted from Leask et al.’s [4] ‘checklist for reporting intervention co-creation’ and Eyles et al.’s [27] amended version of a checklist for reporting non-pharmacological interventions

### Strategy for data analysis

The PRISMA-ScR will be used to guide the reporting of the scoping review [28]. Whilst, the synthesis of the results from included sources of evidence is more appropriately done with a systematic review, the analysis of data in scoping reviews is generally descriptive in nature [29]. A narrative summary of extracted data will be produced along with the tabulated and/or charted results described in relation to the review question and objectives. Descriptive techniques, such as basic coding of data to particular categories, are recommended as a useful approach when the purpose is to identify concepts or key characteristics related to the concept [20]. Data will be analysed using the well-established method of thematic analysis [30]. This method is characterised by identifying and reporting recurring themes within the data and is a suitable analytic method because it allows for patterns of experience to be recorded, such as understanding adults’ experiences of participating in co-creation. We intend to extract relevant co-creation experience data from the result sections of articles, including verbatim participant quotations. For quantitative data, such as questionnaires, we will attempt to extract the item statements and code them alongside the qualitative data.

## Discussion

The purpose of this scoping review is to uncover the breadth of existing empirical research on co-creation experience with a focus on emotional aspects and from a psychological perspective. An increased understanding of the role of experiences of participating in co-creation processes may help to inform the development and use of co-creation processes and lead to potential benefits for the co-creators’ and co-created outcome.

This scoping review has some limitations, which reflect the balance between conducting a wide search to discover the breadth of existing literature and the pragmatic constraints of conducting the review. This scoping review searches for published peer-reviewed work from SCOPUS and the Health CASCADE Co-creation Database. Other databases could be searched but for pragmatic reasons, these two databases were selected for their breadth and relevancy. Another limitation is that it was necessary to restrict the search terms for capturing ‘co-creation’ for the search in Scopus to maintain a manageable number of records retrieved to screen by the research team. However, authors may use different terms or descriptions. For instance, variations of terms like co-creation, co-design, and co-production, whether written with a dash or space can affect the number of articles retrieved. Boundaries on the search terms relating to experience were also formed, for example, specific emotions were not included in the search string, due to the large range of possible emotions that can be experienced, which would make the search unwieldy. We also have not used any of the advanced search features of the databases, such as proximity searching, which could potentially improve the specificity.

A strength of this review is the comprehensive snowballing search strategy to capture additional relevant papers. The results will be submitted to a peer-reviewed journal and to scientific conferences. The plan for dissemination includes digital science communication platforms and presentations.

## Supplementary Information

Additional file 1: PRISMA-P 2015 Checklist.

Additional file 2: Search Strategy–Scopus.

## Data Availability

Not applicable.

## Abbreviations

AI: Artificial intelligence
PRISMA-P: Preferred reporting items for systematic review and meta-analysis protocols
PRISMA-ScR: Preferred reporting items for systematic review and meta-analysis–extension for scoping reviews
