# Supplementary material for: Adult co-creators’ emotional and psychological experiences of the co-creation process: a Health CASCADE scoping review protocol
Source: Syst Rev. 2024 Sep 11;13:231. doi: 10.1186/s13643-024-02643-9 (PMC11389324; doi:10.1186/s13643-024-02643-9)
Supplement: Supplementary file 2 — Additional file 2: Search Strategy–Scopus. [file 13643_2024_2643_MOESM2_ESM.docx]

Additional file 2

Search Strategy – Scopus

| Search within article title, abstract, keywords | Query | Limiters applied |
| --- | --- | --- |
| #1 | "co-creat*" OR "co-production" OR "co-design" OR "experience based design" |  |
| #2 | “experien*” OR “emotion*” OR “psycholog*” OR “mental state*” OR “positive affect” OR “negative affect” OR “affective state*” OR “affective response*” OR “feeling*” OR “empower*” OR “sociali*” OR “autonomy” OR “competency” OR “competence” OR “relatedness” |  |
| #3 | #1 AND #2 | English, Spanish, Dutch, Chinese, French Language only.  Exclude document type: Conference paper, conference review, book chapter, book, review, note, editorial, short survey, letter, erratum, retracted.  Include from year 1970. |

*Note*: * is a wild card character that may be used in place of any number of characters in a search word
